# Velocity-aware spatial-temporal attention LSTM model for inverse dynamic model learning of manipulators

**DOI:** 10.3389/fnbot.2024.1353879

**Published:** 2024-02-09

**Authors:** Wenhui Huang, Yunhan Lin, Mingxin Liu, Huasong Min

**Affiliations:** Institute of Robotics and Intelligent Systems, Wuhan University of Science and Technology, Wuhan, Hubei, China

**Keywords:** manipulators dynamics, model learning, long short-term memory network (LSTM), spatial-temporal attention, velocity aware

## Abstract

**Introduction:**

An accurate inverse dynamics model of manipulators can be effectively learned using neural networks. However, further research is required to investigate the impact of spatiotemporal variations in manipulator motion sequences on network learning. In this work, the Velocity Aware Spatial-Temporal Attention Residual LSTM neural network (VA-STA-ResLSTM) is proposed to learn a more accurate inverse dynamics model, which uses a velocity-aware spatial-temporal attention mechanism to extract dynamic spatiotemporal features selectively from the motion sequence of the serial manipulator.

**Methods:**

The multi-layer perception (MLP) attention mechanism is adopted to capture the correlation between joint position and velocity in the motion sequence, and the state correlation between hidden units in the LSTM network to reduce the weight of invalid features. A velocity-aware state fusion approach of LSTM network hidden units' states is proposed, which utilizes variation in joint velocity to adapt to the temporal characteristics of the manipulator dynamic motion, improving the generalization and accuracy of the neural network.

**Results:**

Comparative experiments have been conducted on two open datasets and a self-built dataset. Specifically, the proposed method achieved an average accuracy improvement of 61.88% and 43.93% on the two different open datasets and 71.13% on the self-built dataset compared to the LSTM network. These results demonstrate a significant advancement in accuracy for the proposed method.

**Discussion:**

Compared with the state-of-the-art inverse dynamics model learning methods of manipulators, the modeling accuracy of the proposed method in this paper is higher by an average of 10%. Finally, by visualizing attention weights to explain the training procedure, it was found that dynamic modeling only relies on partial features, which is meaningful for future optimization of inverse dynamic model learning methods.

## 1 Introduction

The most important issue of manipulator dynamics is how to build an inverse dynamics model to provide joint torques/forces in terms of the configuration (joint positions, velocities, and accelerations) of manipulators (Baressi Šegota et al., 2020; Liu Z. et al., 2022). With the increasing complexity of tasks required for high-dimensional manipulators, precise, and stable inverse dynamics modeling methods are increasingly needed for manipulators motion control.

The dynamics modeling methods for manipulators can be divided into the white-box method, black-box method, and gray-box method (Geist and Trimpe, 2021; Liu et al., 2023). The white-box method, also known as the analytical modeling method, obtains the dynamics model of a manipulator based on theoretical modeling and parameter identification. It has higher transparency and interpretability. However, due to factors such as friction and transmission clearance that are difficult to model, it leads to low accuracy. In general terms, the black-box method refers to machine learning models. It can learn dynamics characteristics that cannot be accurately modeled by white-box methods, from data to obtain higher accuracy models (Williams and Rasmussen, 1995). Gray-box method, also known as the hybrid modeling method, is a fusion of the black-box method and white-box method. It combines the non-linear modeling ability of the black-box method with the interpretability of the white-box method. At present, gray-box methods rely more on the modeling accuracy of white-box methods (Çallar and Böttger, 2022; Reuss et al., 2022), but it has not yet reached the level where the advantages of both white-box and black-box methods can be fully utilized. The authors believe that compared with other fields, the problems of black-box methods in inverse dynamics modeling of manipulators have not been fully discussed. Given this, the research of this work focuses on how to use black-box methods for learning inverse dynamic models.

Deep neural network learning methods have achieved great success in fields such as image processing and natural language processing, but their application research in the field of manipulators dynamics is slightly lagging behind. Machine learning methods that have been used for inverse dynamics model learning of manipulators include GPR (Gaussian process regression) (Williams and Rasmussen, 1995), LWPR (locally weighted projection regression) (Vijayakumar et al., 2005), MLP (Multi-layer Perception) (Yilmaz et al., 2020), GRU (Gated Recurrent Unit) (Cho et al., 2014), and LSTM (Long Short-Term Memory) (Greff et al., 2016). GPR is a supervised learning method used to solve regression and classification problems (Seeger, 2004; Nguyen-Tuong et al., 2009), which optimizes hyperparameters by maximizing the likelihood and can adapt to different datasets more quickly (Rueckert et al., 2017). However, GPR has high computational complexity and takes too long to train on large datasets. Compared to GPR's precise inference with a computational complexity of *O*(*n*^3^), LWPR's computational complexity is *O*(*n*), but it requires manual adjustment of a considerable number of hyperparameters. Yilmaz et al. (2020) use the MLP network to learn the inverse dynamics model of the da Vinci surgical robot and estimate the external forces at the end-effect, this method achieves high accuracy in estimating contact forces within a local range. If the robot is running in an unknown space or a higher speed motion mode, using MLP networks to predict contact forces will result in significant prediction errors. Due to the lack of modeling of temporal features, this method is difficult to obtain an accurate and highly generalized inverse dynamics model. Recurrent neural networks are often used to handle sequential data for time series prediction tasks. Rueckert et al. (2017) use LSTM networks to learn the inverse dynamic model of robot manipulator. Compared to methods such as GPR, LSTM can achieve higher modeling accuracy with a low computational complexity of only *O*(*n*). Mukhopadhyay et al. (2019) have conducted experimental comparative analysis on RNN, LSTM, GRU, and MLP, and the analysis results show that LSTM and GRU can better extract long and short-term features in sequences compared to other methods.

Most sequence modeling methods only emphasize the dependency relationships between sequence nodes, while ignoring other correlations, such as spatiotemporal correlations (Kong and Wu, 2018). Spatiotemporal correlation plays a crucial role in many applications, such as crime prediction (Xia et al., 2022), traffic prediction (Karim et al., 2022), behavior prediction (Song et al., 2018), etc. The spatiotemporal attention mechanism can more accurately capture the temporal and spatial distribution characteristic of the target occurrence.

A dynamic temporal attention (DTA) network has been proposed to achieve early prediction of traffic accidents based on driving recorders, and the network can learn to select discriminative temporal segments of a video sequence with the DTA module (Karim et al., 2022). Song et al. (2018) designed a spatiotemporal attention mechanism combined with long short-term memory networks to recognize and detect human 3D actions from bone data. The temporal attention weights of this work are calculated by the input features, but it ignores the correlation between the hidden layer states in the long short-term memory network. An interpretable spatiotemporal attention long short-term memory model (STA-LSTM) based on a dynamic attention mechanism was proposed for flood prediction (Ding et al., 2020). The spatiotemporal attention weights are calculated based on input features and the hidden layer state of the long short-term memory network, which better utilizes the hidden layer state of LSTM. Du et al. (2018) proposed a spatiotemporal attention mechanism based on effective interactive perception self-attention to recognize human actions in RGB images and capture the interaction characteristics between local features. The above approaches all apply the spatiotemporal attention mechanism to sequence modeling, relying on massive data. We also need to conduct an in-depth analysis of the characteristics of the robot motion sequence and improve the feature extraction algorithm to better apply these methods to the inverse dynamics modeling of manipulators.

The data sequence of manipulators used for inverse dynamics model is different from videos or other sequences, as it is rich in noise, misaligned in timing, and has a small amount of data. It is difficult to achieve ideal results by directly applying spatiotemporal attention mechanisms. To accurately calculate the spatiotemporal attention weights, it is possible to consider using a multi-layer perceptron (MLP) feedforward attention mechanism for spatiotemporal attention. Zhang et al. (2020) analyzed the difference between the self-attention mechanism and the feedforward attention mechanism. Compared to the self-attention mechanism, the feedforward attention mechanism can be seen as pure “monotonic left-to-right diagonal attention,” it restricts attention to the diagonal instead of obtaining contextual information. For the inverse dynamics model learning network of a manipulator, (1) the input only includes changes in past motion sequences, this is one of the reasons for adopting a feedforward attention mechanism. (2) Moreover, compared to self-attention mechanism, using the feedforward attention mechanism can reduce some parameters and effectively reduce computational complexity. (3) The single-layer feedforward attention mechanism lacks modeling ability in complex scenes, while multi-layer perceptron has stronger modeling ability. (4) Using the multi-layer perceptron feedforward attention mechanisms at the input and output ends of LSTM helps to better extract spatial features of input data and temporal features in the hidden layers of LSTM.

Usually, the acceleration of a manipulator is not directly obtained, but rather derived from the differentiation of velocity. Therefore, velocity is particularly important for predicting joint forces and torques. In addition to the four suggestions at the end of the previous paragraph, to better learn the temporal characteristics of time series from LSTM hidden states, it is also necessary to design a velocity-aware LSTM hidden layer state fusion method. The commonly used temporal attention can distinguish the importance of hidden layer states at different time steps, which is very effective for learning models of temporal sequences with obvious regularity. However, for complex and small data temporal sequences, it is difficult to train a suitable model using this kind of method. In another approach, the attention weight of the last layer (usually called *h*_*n*_) of the LSTM hidden layer is calculated, but this method is prone to problems such as information loss or information attenuation (Tao and Liu, 2018). The attention mechanism includes weighted averaging of all hidden states in the LSTM hidden layer, and Tao and Liu (2018) has achieved very good results in emotion recognition. In Ostmeyer and Cowell (2019), in the author's opinion, this weighted average method limits the scope of attention mechanism usage. They propose a cyclic weighted average method, which improves the applicability of the algorithm but also significantly increases the computational complexity of the network, which is not conducive to multi-step time-series forecasting model training. In response to the characteristics of language emotion recognition, Xie et al. (2019) uses a self-attention mechanism to fuse the states of all hidden layer neurons in the LSTM network, fully utilizing the hidden layer states at each time step of the LSTM network. For the output fusion of the LSTM network, it is necessary to combine these theories with the actual characteristics of manipulator motion. In principle, through the joint velocity we can intuitively obtain some factors such as the changes in the direction of motion of the manipulator joint, the frequency of changes, and other factors, which are the characteristics of joint velocity, these factors are important features in motion sequence data. Therefore, we propose an LSTM temporal feature fusion method with a joint velocity aware algorithm, to better extract temporal features contained in LSTM hidden layer states, and improve the accuracy of inverse dynamics modeling of manipulator.

The manipulator dynamics dataset collected from real world has phenomena such as uneven data distribution, rich noise, and small sample size, which can cause problems such as vanishing or exploding gradients, slow convergence, etc. (Sheng et al., 2023). Using residual LSTM networks would be a good choice. Appropriate residual connections can resolve gradient vanishing or exploding, accelerate network convergence, and enhance the network's expressive power.

In summary, the objective of this study is to design a residual LSTM network architecture that integrates spatiotemporal attention mechanisms for accurate estimation of manipulator joint torques. The proposed approach leverages a multi-layer perceptron feedforward attention mechanism to calculate spatiotemporal attention weights. Furthermore, a velocity perception method is devised to effectively combine the hidden units' states of the LSTM network. According to the training method in Yilmaz et al. (2020), a separate network is trained for each manipulator joint to facilitate comparison and analysis. Due to significant quantization noise in the acceleration signals in the dataset and difficulties in physical sampling, the network training in this work does not use acceleration signals. The main contributions of this work are as follows:

I. We propose a residual LSTM network structure combined with a spatiotemporal attention mechanism, which effectively improves the accuracy of inverse dynamics modeling for manipulators.
II. Design a multi-layer perceptron feedforward attention mechanism to calculate spatiotemporal attention weights. The multi-layer perception function better extracts the interactive features between each joint motion, forming more effective attention weights.
III. Design a velocity-aware method to fuse the hidden layer states of LSTM networks and improve the temporal modeling ability of the network.
IV. This work visualizes attention weights and proves that there are differences in the importance of spatiotemporal features at different joints, providing interpretability for network training.

The rest of this paper is organized as follows. Section 2 provides a mathematical formulation of the inverse dynamics model and basic expressions of the model learning. The structure of Velocity-Aware Spatial-Temporal Attention Residual Long Short-Term Memory neural network (VA-STA-ResLSTM) is described in Section 3. Section 4 presents the experimental comparison results, and explains the training procedure by visualizing attention weights. The paper concludes in Section 5.

## 2 Mathematical modeling of manipulators dynamics and problem description

A general dynamics model for an *n*-links serial manipulator can be given by Rueckert et al. (2017) as shown in Equation (1).


(1) $$M\left(q\right)\overset{..}{q}\;+\;V\left(q,\dot{q}\right)\;+\;G\left(q\right)+F_{r}\left(\dot{q}\right)\;=\;\tau \;+\;\tau_{e}$$

Where, *M*(*q*)∈*R*^*n*×*n*^ denotes the inertial matrix, $V\left(q,\overset{•}{q}\right)\in R^{n\times n}$ denotes the Coriolis/centripetal matrix, *G*(*q*)∈*R*^*n*^represents the gravity vector, $F_{r}\left(\overset{•}{q}\right)\in R^{n}$ accounts for the non-linear force and unmodeled force (such as friction effects), τ∈*R*^*n*^ is the vector of input torques acting at the joints, $\tau_{e}\in R^{n}$ represents any bounded external forces/torques caused by the environment, $q,\overset{•}{q},\ddot{q}\in R^{n}$ are the joint positions and their temporal derivatives.

Traditional dynamics models usually use linear approximation models to represent complex non-linear friction and unmodeled forces, which cannot fully describe the physical characteristics of the manipulator dynamics model and bring significant errors to joint torque estimation.

Rueckert et al. (2017) defines the problem of learning the inverse dynamics model of a manipulator as


(2) 𝕐 = f(𝕏)+ξ

Where *𝕐* is the estimated joint torques, *𝕏* is the motion state of the manipulator—configuration, $𝕏={\left[q,\overset{•}{q},\ddot{q}\right]}^{T}$, ξ is gaussian random noise with mean 0 and variance σ_*y*_, *f* represents the function that needs to be modeled.

Construct datasets according to Equation 2, *t* is the time steps, dataset *D* = < *𝕏*_*T*_, *𝕐*_*T*_>, *T* = 1, …, *t*, ${𝕏}_{T}\in R^{2\times n\times t}$ represents the motion data of *n* links serial manipulator, Including joint position and velocity, ${𝕐}_{T}\in R^{n\times t}$ represents joint torque (training a model separately for each joint). In Rueckert et al. (2017), the mean square error loss function of the training model can be described as


(3) $$MSE\;=\;\frac{1}{t\times n}\sum_{i\;=\;1}^{t}\sum_{j\;=\;1}^{n}{\left({\hat{y}}_{i}^{\left[j\right]}-{\tilde{y}}_{i}^{\left[j\right]}\right)}^{2}$$

where ${\hat{y}}_{i}^{\left[j\right]}$ is the actual torque value of joint *j* at time *i*, ${ỹ}_{i}^{\left[j\right]}$ for estimated torque values of joint *j* at time *i*.

The inverse dynamics modeling of manipulators can be described as a multi-time series forecasting problem with multiple variables. The high dimensionality brought by multivariable and multi-time steps results in a large number of features in the dataset with low correlation to the inverse dynamics model, this will directly affect the modeling accuracy and generalization performance of the network.

## 3 Velocity aware spatial-temporal attention residual LSTM network design

**Figure 1:**
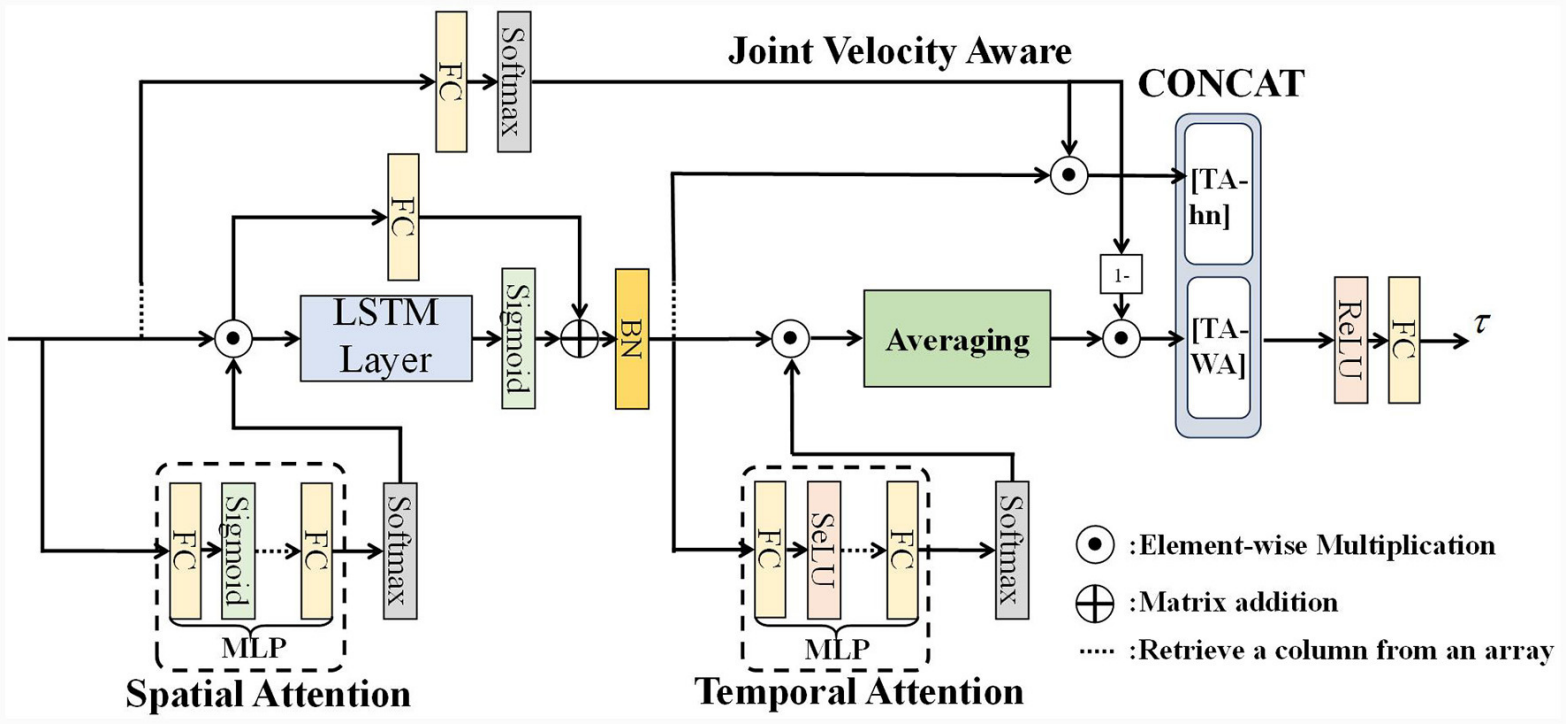
Residual spatial-temporal attention LSTM network structure.

We propose a Velocity Aware Spatial-Temporal Attention Residual Long Short-Term Memory neural network (VA-STA-ResLSTM) to learn Inverse dynamics model of the manipulator. Figure 1 shows the overall framework of the VA-STA-ResLSTM neural network, which includes spatial attention mechanism, temporal attention mechanism, residual LSTM, and joint velocity aware temporal feature fusion method. Although the proposed network structure adds multiple layers of perceptrons, the computational complexity remains O(n).

Next, we will introduce three modules of VA-STA-ResLSTM in sequence: residual LSTM network, spatial attention mechanism, and velocity-aware based temporal attention mechanism.

### 3.1 LSTM network with residual connections

**Figure 2:**
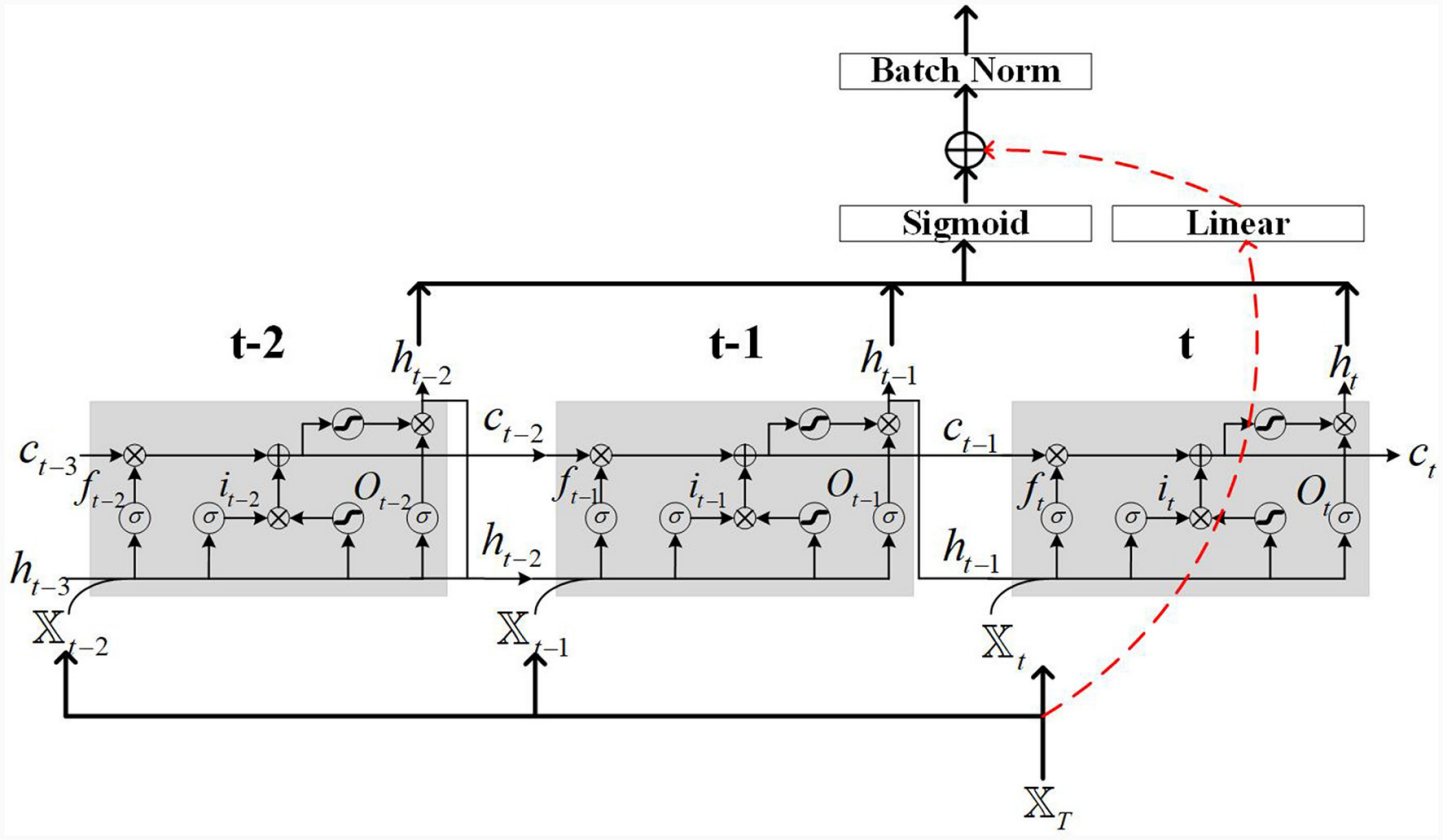
Illustration example of LSTM cells with residual connection.

The network structure of the residual LSTM used in this paper is shown in Figure 2. Figure 2 depicts an LSTM network with residual connections, which consists of original LSTM neurons. The residual connection is composed of a linear layer and a Sigmoid activation function. The function of the linear layer is to adjust the matrix dimension of the input features, while the sigmoid activation function is used to improve the non-linear modeling ability of the network. LSTM has a mechanism called a “gate” that selectively remembers or forgets, thereby better capturing long-term dependencies. The primary gates include the input gate (*i*_*t*_), forget gate (*f*_*t*_), and output gate (*O*_*t*_), with *𝕏*_*t*_ denoting the input feature at time step *t*. Each gate is composed of a sigmoid layer and an element-wise multiplication operation.

In Figure 2, the red dashed lines represent the residual connections. The motion of the robot manipulator may have randomness and non-uniformity in its trajectory, which can lead to gradient vanishing or exploding during LSTM network training, using residual connections can solve such problems. The residual connections in Figure 2 employ linear layers to match the matrix size. A sigmoid activation function is applied to the LSTM output to enhance its non-linear modeling capability. Additionally, batch normalization is utilized on the output to prevent model overfitting.

### 3.2 Spatial attention mechanism based on multilayer perceptron

**Figure 3:**
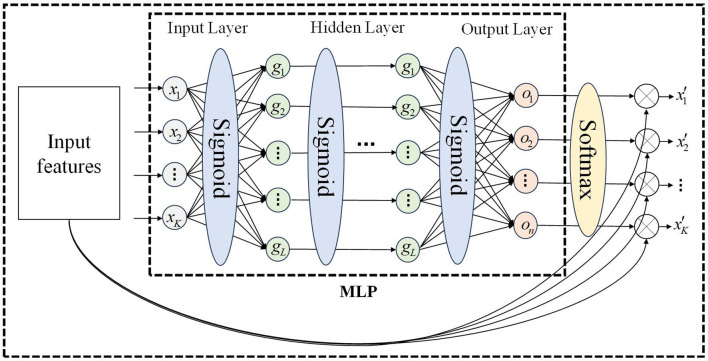
MLP network for spatial attention mechanism.

The motion sequence of the manipulator includes joint position, velocity, and acceleration. Due to the structure of the manipulator, motion, and force are transmitted joint-by-joint, and there is a correlation between the motion characteristics of the front and rear joints. Obtaining the motion correlation between different joints is the key to constructing the spatial attention mechanism. We propose a spatial attention mechanism based on a multi-layer perceptron, which is divided into the input layer, hidden layer, and output layer, as shown in Figure 3. This attention mechanism only relies on the input features to calculate the attention weight vector, and each motion feature is assigned a spatial attention weight, allowing the network to focus on the more important motion feature. Compared to the self-attention mechanism, the attention calculation method of MLP has lower computational complexity, stronger anti-interference ability, and is more suitable for processing serial manipulator motion sequences (Zhang et al., 2020; Liu Y. et al., 2022).

In Figure 3, after batch processing, the input feature matrix is *𝕏*∈*R*^*K*×*d*^, where *K* represents the number of features and *d* is usually smaller than t and is a fixed constant, the output feature matrix is *𝕏*′∈*R*^*K*×*d*^. If the degree of freedom of the manipulator is *n*, the number of input features *K* can be expressed as 2 × *n* (position, velocity) or 3 × *n* (position, velocity, acceleration). The calculation of spatial attention weights is described in Algorithm 1, where *W*_*input*_ represents the weights of the input layer of MLP, and *b*_*input*_ represents the bias of the input layer of MLP, *W*_*hidden*_*i*_ represents the weights of the ith hidden layer of MLP, and *b*_*hidden*_*i*_ represents the bias of the ith hidden layer of MLP, *W*_*output*_ represents the weights of the output layer of MLP, and *b*_*output*_ represents the bias of the output layer of MLP. Input the motion sequence, output spatial attention weights. The dimensions of the output weight matrix and input feature matrix are the same.

**Algorithm 1:**
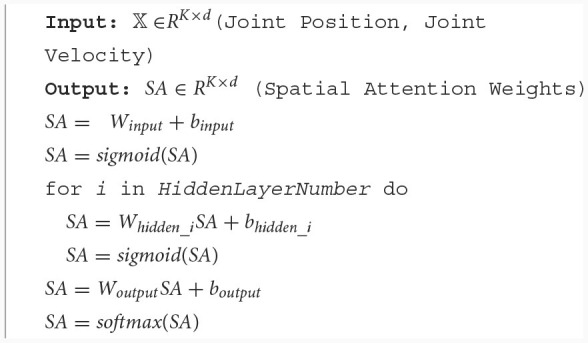
Spatial attention weights calculation.

Where *HiddenLayerNumber* is the number of hidden layers for multi-layer perceptron. For the *k*-th input feature, the softmax function used in the normalization method (*softmax*) (Song et al., 2018) is described as:


(4) $$\alpha_{d,k}=\frac{e^{SA_{d,k}}}{\sum_{i=1}^{K}e^{SA_{d,i}}}$$

Where α_*d, k*_ is a normalization of spatial attention scores, *d* is the total time step, *K* represents the total number of features, and *k* is the *k*-th feature. The larger the calculated weight, the higher the correlation between the feature and the current moment. The smaller the weight, the lower the correlation. By element-wise multiplication, the weighted feature matrix *𝕏*^**′**^ is obtained and input into the residual LSTM network. The calculation of *𝕏*^**′**^ is described as


(5) $${𝕏}^{\prime }=𝕏⊙\alpha$$

Where ⊙ represents element-wise multiplication.

### 3.3 Temporal attention mechanism with velocity aware module

The temporal attention mechanism proposed in this paper mainly consists of two parts. Firstly, it employs a multi-layer perceptron attention mechanism to assign weights to all hidden layer states of the LSTM. Secondly, it incorporates a velocity-aware module to fuse the hidden layer states of the LSTM.

The hidden state of LSTM hidden layer neurons preserves the long-term and short-term temporal characteristics of the model. Generally, temporal attention mechanisms are designed in the temporal direction, which means analyzing which time steps are highly correlated with the output. This approach has achieved very good modeling results, as shown in Song et al. (2018). To design an attention mechanism along the temporal direction, transforming the hidden layer neuron state matrix into a single dimensional vector with the same time step size as the input sequence, *R*^*h*×*d*^→*R*^1 × *d*^, then perform weighted calculations, where *h* is the hidden size of LSTM network. However, for a continuous system, the importance of distinguishing different time step features has not yet been fully utilized by LSTM networks in learning long-term and short-term features, if we only set attention mechanism along the temporal direction, especially on datasets with insufficient changes or irregular changes, which can easily lead to overfitting and poor training effectiveness.

**Figure 4:**
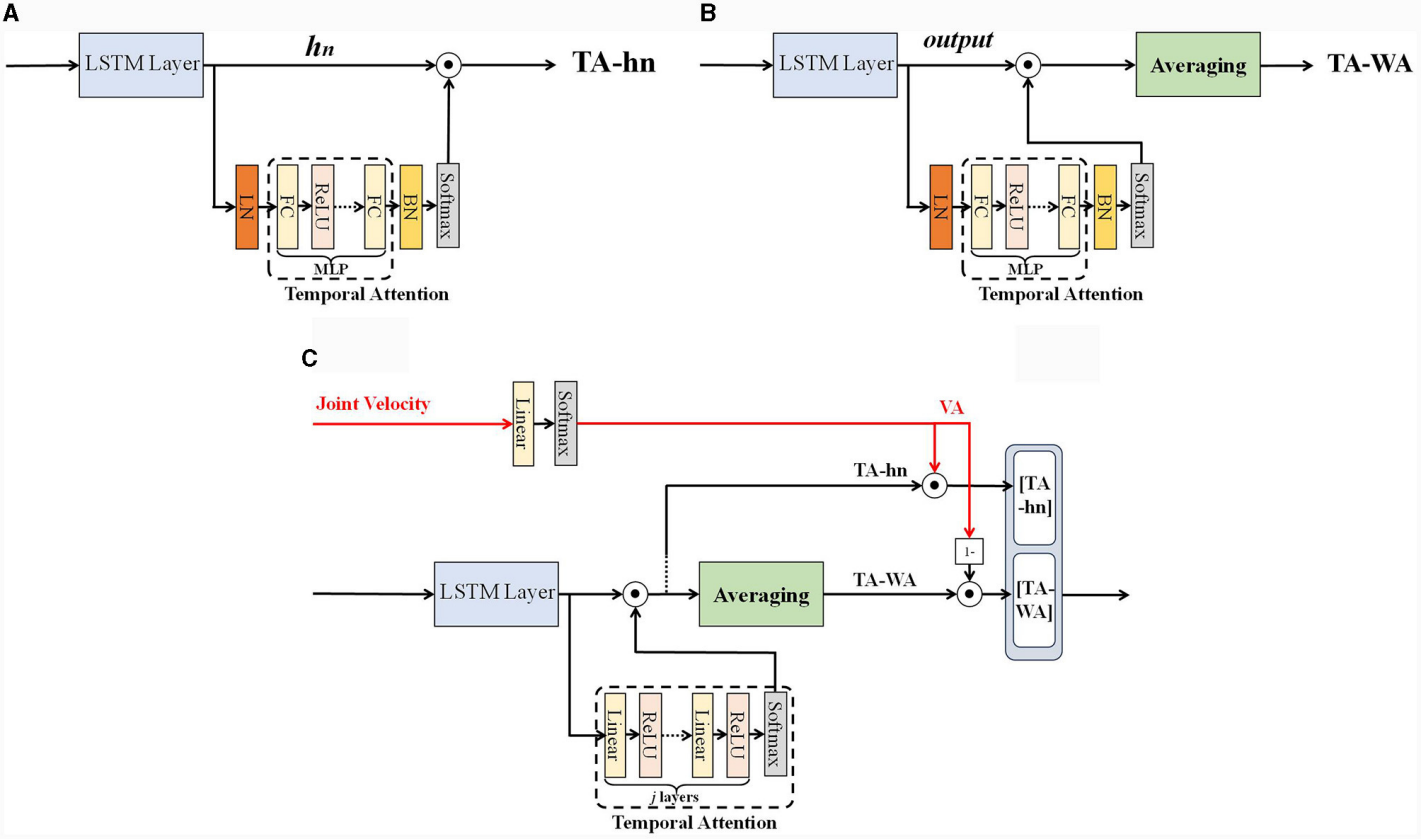
**(A)** TA-hn, **(B)** TA-WA, and **(C)** velocity aware temporal attention (VA-TA).

The number of hidden layer units in each time step of the LSTM network is actually the width of the LSTM network, which represents the capacity of the LSTM network. In this work, we can define it as the feature direction. Calculating along the feature direction transforms the hidden layer neuron state matrix into a single dimensional vector with the same number of hidden layer units at each time step. Unlike the time direction, attention weights calculation can be directly performed along the feature direction by selecting the last hidden layer state (Du et al., 2018), as shown in Figure 4A. In Figure 4A, the *h*_*n*_ represents the last hidden layer state of LSTM. *A*∈*R*^1 × *h*^ represents the time attention weight matrix, calculate attention weights: $A\times h_{n}\in R^{1\times h}$. Due to *h*_*n*_ contains information about the entire input sequence and can be applied to various tasks, but it is easy to ignore the temporal characteristics of each time step. Another way is to achieve attention weights by weighting and averaging all hidden layer states (Tao and Liu, 2018), as shown in Figure 4B. In Figure 4B, the *output* represents all hidden layer states of LSTM. Calculate attention weights: *mean* (*A*×*O*)∈*R*^1 × *h*^, where *O*∈*R*^*d*×*h*^ stands for all hidden layer states, *A*∈*R*^*d*×*h*^ represents the time attention weight matrix. This method adopts a weighted average approach for attention weight calculation, and the network is more sensitive to sudden changes in the temporal characteristics of each time step, but it is also prone to prediction bias. For the convenience of description, this paper names the first method as **TA-hn** (Temporal Attention with *h*_*n*_) and the second method as **TA-WA** (Temporal Attention with Average), as shown in Figure 4. A multi-layer perceptron attention mechanism is used to assign weights to the hidden layer states of all LSTM neurons. Subsequently, TA-WA and TA-hn are calculated separately. These values, TA-WA and TA-hn, will serve as inputs for the velocity-aware fusion module.

We have designed a fusion method for velocity-aware temporal features, taking into account the advantages of both **TA-hn** and **TA-WA**, as shown in Figure 4C. In Figure 4C, Our method is called as VA-TA (Velocity Aware Temporal Attention), the **VA** represents the normalized score calculated from joint velocities. The **[TA-hn]** represents weighted **TA-hn**, and the **[TA-WA]** represents weighted **TA-WA**. Firstly, extract the velocity feature vector of the joint from the feature matrix input into the network. Then, the fusion weights are calculated using the fully connected layer and *softmax* function. The weights calculated using **TA-hn** and **TA-WA** methods have complementarity therefore, the calculation of attention weights is designed in a complementary form. In the VA-TA algorithm, the value of VA is dynamically adjusted based on joint velocity. A higher VA value corresponds to a greater proportion of **TA-hn** in the fusion, while a lower VA value corresponds to a greater proportion of **TA-WA** in the fusion. The specific process is as shown in Algorithm 2, where *output* represents all hidden states of the LSTM network's hidden layer, *h* represents the number of neurons in the LSTM hidden layer, *s* represents the sequence length of input features, *W*_*va*_ represents the weights of the network, and *b*_*va*_ represents the bias of the network.

**Algorithm 2:**
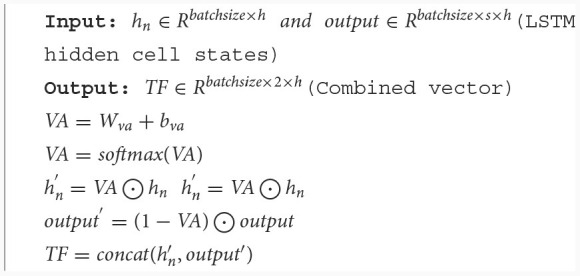
Velocity aware based temporal feature fusion.

**Figure 5:**
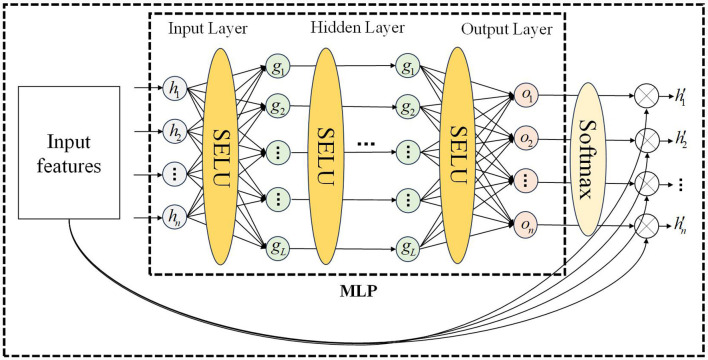
Temporal attention mechanism MLP structure.

Prior to employing velocity-aware fusion, we developed a multi-layer perceptron temporal attention mechanism to filter the LSTM hidden layer states, as shown in Figure 1. The temporal attention mechanism designed in this paper is a multi-layer perceptron, as shown in Figure 5, and the calculation process is shown in Algorithm 3, where *W*_*input*_ represents the weights of the input layer of MLP, and *b*_*input*_ represents the bias of the input layer of MLP, *W*_*hidden*_*i*_ represents the weights of the *i*th hidden layer of MLP, and *b*_*hidden*_*i*_ represents the bias of the *i*th hidden layer of MLP, *W*_*output*_ represents the weights of the output layer of MLP, and *b*_*output*_ represents the bias of the output layer of MLP. Among them, two output weighting methods are described, include the state of the last hidden layer and all hidden layer states. *HiddenLayerNumber* is the number of hidden layers for multi-layer perceptron.

**Algorithm 3:**
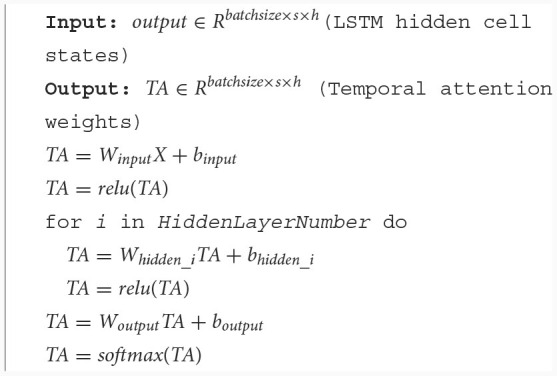
Temporal attention.

Finally, calculate the weighted hidden layer weight vector as shown in Equation (6).


(6) $${ℍ}^{\prime }=\text{TA}⊙ℍ$$

Where ℍ is the set of the original LSTM hidden units' states, *TA* is the calculated weighted score of the temporal attention, ℍ∈*R*^*s*×*k*^, ℍ′ is the weighted hidden units' states.

## 4 Experiments and results

Experimental verification is conducted on three datasets. The open dataset in Polydoros et al. (2015) is named KUKA-I, the open dataset in Meier et al. (2014) is named KUKA-II, and the self-built dataset UR5-data. These datasets are collected on real-world manipulators, with training and testing sets set to (16,000, 4,000), (13,000, 4,560), and (15,000, 5,000), respectively, and the data is processed using a Butterworth low-pass filter. The data normalization technique employed makes use of the StandardScaler function from the *sklearn* library. This normalization technique targets the mean and variance of each individual dimension rather than the entire datasets. In addition, methods such as fuzzy similarity calculation (Versaci et al., 2022) can be used during preprocessing to reduce computational complexity, but this issue is not discussed in this article.

**Figure 6:**
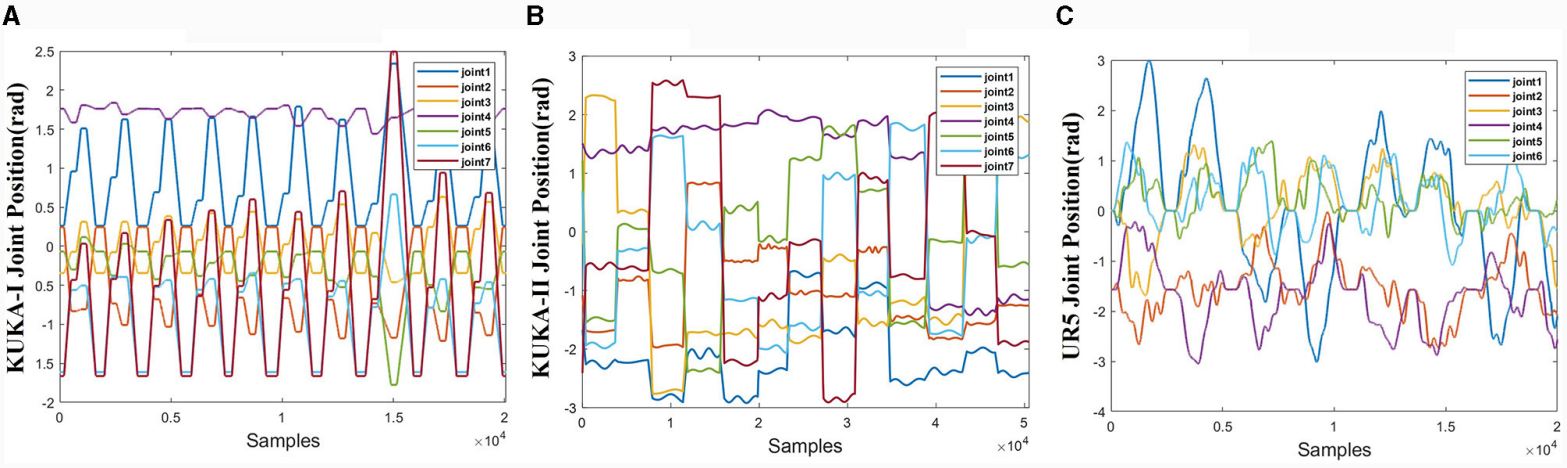
Joint trajectories of the three datasets. **(A)** KUKA-I. **(B)** KUKA-II. **(C)** UR5-data.

The joint trajectories of the three datasets are shown in Figure 6. The trajectories of the three datasets exhibit different characteristics. The KUKA-I dataset exhibits periodicity, the KUKA-II dataset exhibits non-periodic and gentle motion, and the UR5-data dataset exhibits rapid and irregular motion.

The computer used in the experiment is a GPU, RTX3060Ti, with a software architecture of PyTorch and an optimizer of Adam. The batch size is set to 128. The learning rate is set to dynamic adjustment mode, with an initial value of 0.01. The learning rate is gradually reduced based on training loss to achieve stable convergence. All model training and testing use the mean square error given in Equation 3, which can intuitively and accurately represent the difference in model accuracy.

We conduct three sets of experiments: (a) For the network structure proposed in this paper (Figure 1), different combinations of hidden layer layers, activation function, residual connections, and different temporal attention mechanisms are set, and ablation experiments are conducted on three datasets to obtain the optimal combination of network structures. (b) The best combination network proposed in this article is compared with SOTA's inverse dynamics model learning method of serial manipulator, to prove the progressiveness of the method proposed in this article. (c) We visualize attention weights in the neural network, analyze the characteristics of the network model learning process, and explain the training process.

### 4.1 Ablation

To verify the effectiveness of the proposed method, different network structures are compared. To analyze the impact of different numbers of spatiotemporal attention hidden layers, the combination of activation functions, the presence or absence of residual connections, and the presence or absence of velocity aware modules on model accuracy.

**Figure 7:**
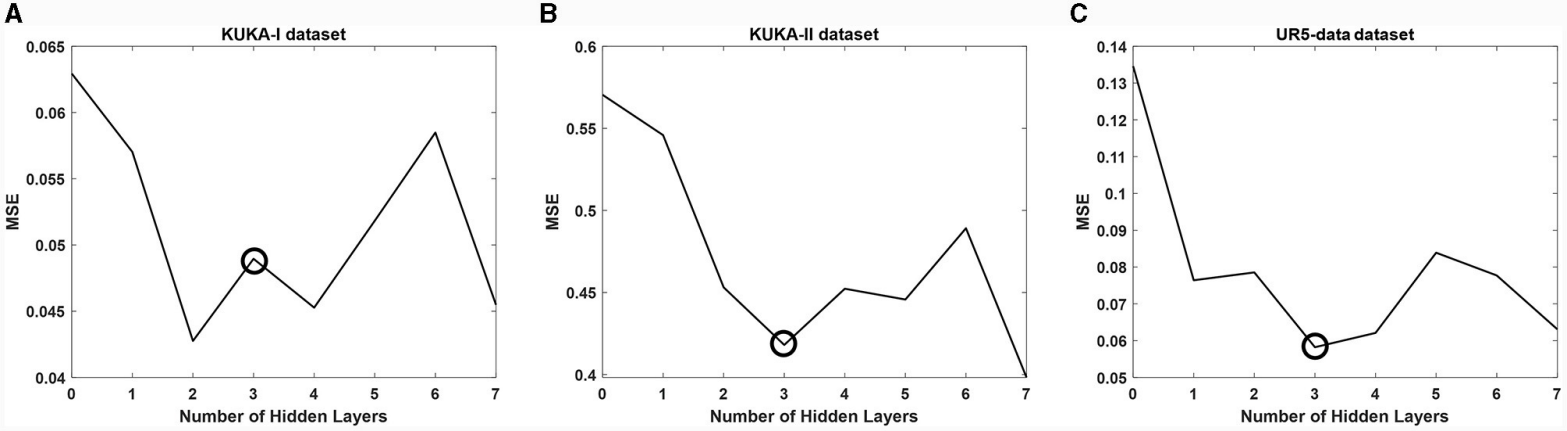
The impact of different numbers of hidden layers of spatial attention and temporal attention on average estimation accuracy. **(A)** KUKA-I dataset, **(B)** KUKA-II dataset, and **(C)** UR5-data dataset.

We first adopted a network (VA-STA-ResLSTM) structure described in Section 3 and compared different numbers of hidden layers of spatial attention and temporal attention on three datasets. To ensure a fair comparison, the number of hidden layers is kept consistent for both spatial attention and temporal attention. The experimental results are shown in Figure 7. As the number of hidden layers increases, the torque estimation accuracy of the model does not necessarily decrease. After setting the number of hidden layers to 3, the model accuracy will show an oscillating trend, but it has already met the acceptable accuracy requirement (Stathakis, 2009). Therefore, subsequent experiments will be conducted under the condition of three hidden layers.

**Figure 8:**
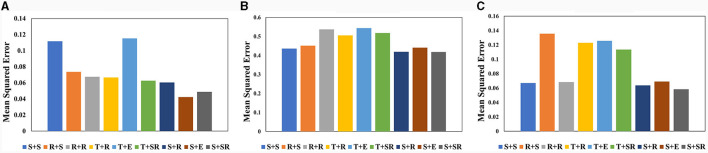
The comparison of the average mean square error of joint torque estimation obtained from VA-STA-ResLSTM network with different combinations of spatial attention and temporal attention activation functions tested on three datasets. **(A)** KUKA-I. **(B)** KUKA-II. **(C)** UR5-data.

Although there has been thorough research on the impact of different activation functions on network performance, we still conduct different combination tests of spatial attention and temporal attention activation functions. This paper conducts a Combination test of activation functions on three datasets on VA-STA-ResLSTM network. We tested two main types of activation functions: Sigmoid functions, *ReLU* and its improved versions. Sigmoid function Classes include the *Sigmoid* function and *Tanh* function, while *ReLU* and its improvements include *ReLU* function, *ELU* function, and *SELU* function. The comparison of the average mean square error of joint torque estimation obtained from different combinations of activation functions is shown in Figure 8. The a+b Form represents the combination of activation functions, where “a” is the activation function used by the spatial attention mechanism and “b” is the activation function used by the temporal attention mechanism. In Figure 8, the *Sigmoid* function is denoted as S, the *Tanh* function is denoted as T, the *ReLU* function is denoted as R, the *ELU* function is denoted as E, and the *SELU* function is denoted as SR. According to the test results, S+SR is the optimal combination among them, and high-precision torque estimation models can be obtained in all three datasets. Subsequent experiments will use the S+SR activation function combination.

After determining the number of hidden layers of attention mechanism and the combination of activation functions, we conduct comparative tests on three datasets based on the network structure and different attention weight calculation methods described earlier in this article.

The experimental results are shown in Tables 1–3. **LSTM+SA** represents an LSTM network with a basic spatial attention mechanism described in Section 3.2, **ResLSTM** represents the LSTM network with residual connections, **TA-hn** and **TA-WA** are the weights calculation methods for the temporal attention mechanism introduced in Section 3.3, **VA-TA** is a velocity aware time feature fusion method proposed in this article. It can be seen that the combination proposed in this article (**ResLSTM+SA+VA-TA**) can achieve the optimal modeling accuracy. The time feature fusion of velocity perception better utilizes the LSTM hidden layer state and compensates for the problem of modeling accuracy changes in different datasets using one single method. Residual connections can not only further improve the learning accuracy of the model, but also can solve the problems of vanishing or exploding gradients.

**Table 1:** Normalized mean square error of joint torque estimation obtained from testing on the KUKA-I dataset.

**Network**	**Joint 1**	**Joint 2**	**Joint 3**	**Joint 4**	**Joint 5**	**Joint 6**	**Joint 7**	**MSE**
ResLSTM+SA+TA_hn	0.1257	0.0027	0.0237	0.0294	0.0981	0.1857	0.0181	0.069057143
ResLSTM+SA+TA_WA	0.1331	0.0028	0.0178	0.017	0.0855	0.1196	0.0193	0.056442857
LSTM+ SA+ VA-TA	0.0737	0.0077	0.0105	0.0368	0.09	0.1979	0.0254	0.06314
**ResLSTM+SA+** **VA-TA**	0.0655	0.006	0.0123	0.0449	0.0405	0.1491	0.0244	**0.048957143**

Bold value represents the optimal result.

**Table 2:** Normalized mean square error of joint torque estimation obtained from testing on the KUKA-II dataset.

**Network**	**Joint 1**	**Joint 2**	**Joint 3**	**Joint 4**	**Joint 5**	**Joint 6**	**Joint 7**	**MSE**
ResLSTM+SA+TA_hn	0.6599	0.858	0.3201	0.4694	0.575	0.1506	0.2833	0.4737
ResLSTM+SA+TA_WA	0.5018	0.5115	0.6525	0.5054	0.6728	0.1618	0.4054	0.4873
LSTM+ SA+ VA-TA	0.2935	0.6032	0.3968	0.5822	0.5159	0.1863	0.1655	0.3919
**ResLSTM+SA+** **VA-TA**	0.2937	0.7959	0.3482	0.5021	0.5885	0.1337	0.2642	**0.4180**

Bold value represents the optimal result.

**Table 3:** Normalized mean square error of joint torque estimation obtained from testing on the UR5-data dataset.

**Network**	**Joint 1**	**Joint 2**	**Joint 3**	**Joint 4**	**Joint 5**	**Joint 6**	**MSE**
ResLSTM+SA+TA_hn	0.0368	0.0375	0.0631	0.1513	0.1762	0.0433	0.0847
ResLSTM+SA+TA_WA	0.0391	0.0589	0.0775	0.1075	0.1662	0.034	0.0805
LSTM+ SA+TA_VEL	0.0745	–	0.0226	0.0954	0.2826	0.0482	–
**ResLSTM+SA+TA_VEL**	0.0398	0.0169	0.0143	0.0878	0.1554	0.0351	**0.058217**

Bold value represents the optimal result.

### 4.2 Comparison experiment of joint torque estimation accuracy with other types of neural networks

To further illustrate the progressiveness of the proposed method, the optimal combination network obtained above is compared with LSTM (Greff et al., 2016), GRU (Cho et al., 2014), RNN (Mukhopadhyay et al., 2019), MLP (Yilmaz et al., 2020), and Transformer (Çallar and Böttger, 2022) on three different data sets, as shown in Tables 4–6. Compared with the LSTM network, the training results on three datasets show that the proposed network has improved the average estimation accuracy of joint torque by 61.88%, 43.93%, and 71.13%, respectively. Compared to other methods, the average estimation accuracy of joint torque with the proposed method is always optimal.

**Table 4:** Compared with SOTA methods on the KUKA-I dataset.

**Method**	**Joint 1**	**Joint 2**	**Joint 3**	**Joint 4**	**Joint 5**	**Joint 6**	**Joint 7**	**MSE**
LSTM	0.3692	0.0037	0.0511	0.0629	0.0581	0.2891	0.0642	0.1283
GRU	0.0553	0.0014	0.0288	0.0340	0.1237	0.1615	0.0250	0.0613
RNN	0.5203	0.0036	0.0096	0.0270	0.1585	0.5209	0.0272	0.1810
MLP	0.5609	0.0038	0.0152	0.0384	0.0893	0.1510	0.0381	0.1281
Transformer	0.1355	0.0071	0.0741	0.0907	0.1943	0.1799	0.0430	0.1035
**Proposed**	0.0655	0.006	0.0123	0.0449	0.0405	0.1491	0.0244	**0.0489**

Bold value represents the optimal result.

**Table 5:** Compared with SOTA methods on the KUKA-II dataset.

**Method**	**Joint 1**	**Joint 2**	**Joint 3**	**Joint 4**	**Joint 5**	**Joint 6**	**Joint 7**	**MSE**
LSTM	0.5653	0.9119	0.5055	1.5769	0.5360	0.2399	0.8842	0.7456
GRU	0.9200	0.9881	0.4689	0.9454	0.7693	0.2588	0.4943	0.6921
RNN	0.5151	0.9832	0.2465	0.5556	0.6730	0.1922	0.3628	0.5040
MLP	1.2482	1.4420	1.5206	1.1959	1.3976	1.0958	1.1811	1.2973
Transformer	0.5987	0.7193	0.4102	0.7878	0.5468	0.2516	0.5661	0.5543
**Proposed**	0.2937	0.7959	0.3482	0.5021	0.5885	0.1337	0.2642	**0.4180**

Bold value represents the optimal result.

**Table 6:** Compared with SOTA methods on UR5-data dataset.

**Method**	**Joint 1**	**Joint 2**	**Joint 3**	**Joint 4**	**Joint 5**	**Joint 6**	**MSE**
LSTM	0.0531	0.2202	0.1705	0.3826	0.2938	0.0896	0.2016
GRU	0.0465	0.2363	0.0396	0.1765	0.2603	0.0799	0.1398
RNN	0.0277	0.0125	0.0352	0.1168	0.1489	0.0548	0.0659
MLP	0.0779	0.0666	0.204	2.091	0.5167	0.507	0.5772
Transformer	0.0711	0.0654	0.1069	0.1429	0.2348	0.0819	0.1171
**Proposed**	0.0398	0.0169	0.0143	0.0878	0.1554	0.0351	**0.0582**

Bold value represents the optimal result.

### 4.3 Visual explanation of effect of spatial attention weights on learning of inverse dynamics models of robot manipulator

According to the analytical modeling method (Gautier and Venture, 2013), it is known that due to the linear correlation between the mass inertia matrix parameters of the inverse dynamics model, singular value decomposition is required for the regression matrix, and basic dynamic parameters are calculated to characterize the dynamics of the serial manipulator. This indicates that not all motion features are equally important to dynamics.

**Figure 9:**
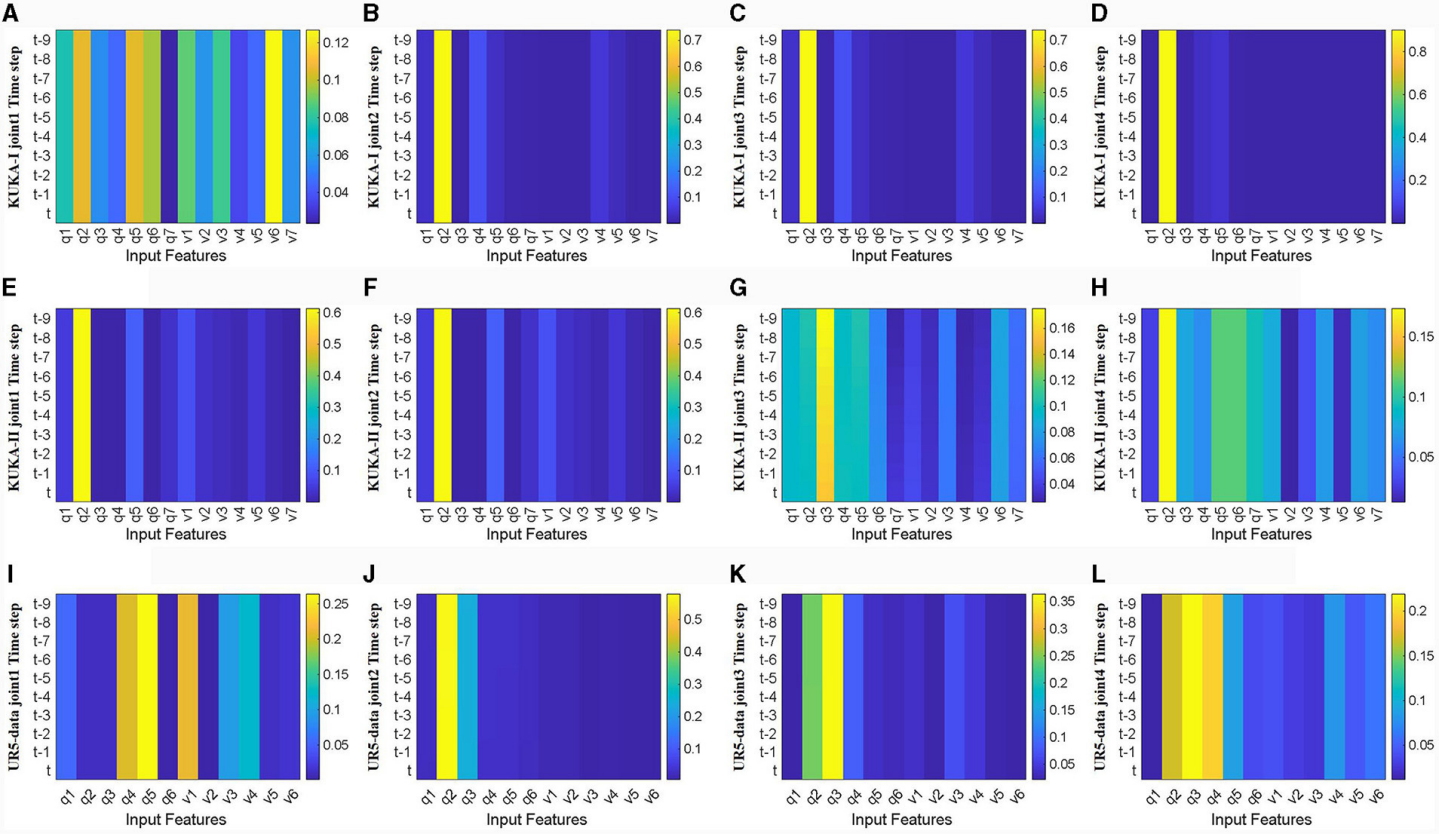
Heat map of spatial attention weights for the first four joints without acceleration feature input (horizontal axis represents input features, *q*1-*q*7 represents positions of each joint, *v*1-*v*7 represents velocities of each joint, vertical axis represents time steps, color changes from blue to yellow, and weights gradually increase). **(A–D)** Represents joints 1 to 4 in the KUKA-I dataset, **(E–H)** represents joints 1 to 4 in the KUKA-II dataset, and **(I–L)** represents joints 1 to 4 in the UR5-data dataset.

Firstly, visualize and analyze the spatial attention weight heatmap described in Section 3.2. The first four joints of the manipulator used in the three datasets are extracted for analysis without acceleration input. As shown in Figure 9, the spatial attention mechanism clearly distinguishes the importance of input features, and for joints with a wider range of motion, joint torque estimation relies on more input features. Comparing the effects of joint position and velocity on attention weights, joint torque estimation output is more dependent on changes in joint position, while the weight heatmap mainly focuses on joint position. This indicates that focusing on changes in the configuration is more conducive to improving the accuracy of inverse dynamics modeling for manipulators.

**Figure 10:**
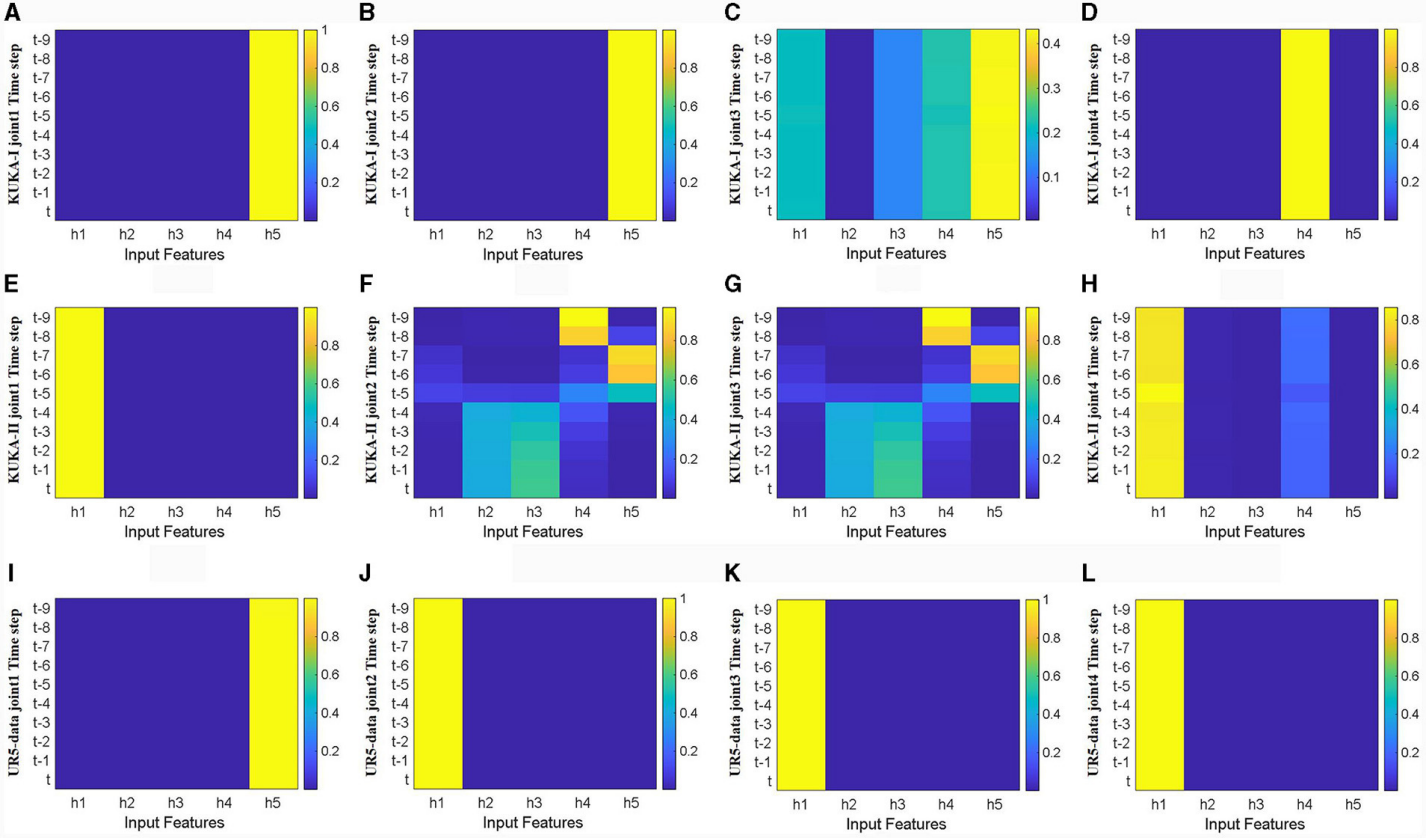
Heat map of temporal attention weights for the first four joints without acceleration feature input (horizontal axis represents hidden layers number, vertical axis represents time steps, color changes from blue to yellow, and weights gradually increase). **(A–D)** Represents joints 1 to 4 in the KUKA-I dataset, **(E–H)** represents joints 1 to 4 in the KUKA-II dataset, and **(I–L)** represents joints 1 to 4 in the UR5-data dataset.

Figure 10 shows the heatmaps of temporal attention weights for different hidden units (*h*_1_, *h*_2_, *h*_3_, *h*_4_, *h*_5_) of LSTM network. The first four joints in three datasets were analyzed without considering acceleration input. The trajectory characteristics of the three different datasets are different. The trajectory characteristics of the KUKA-I dataset are periodic, and the temporal attention weights in Figure 10A are mainly concentrated in the deep hidden layer states. In contrast, the trajectory characteristics of the KUKA-II dataset are non-periodic, and the distribution of temporal attention weights in Figure 10B is irregular. For the UR5 dataset, the trajectory characteristics are non-periodic and the velocity curve is smooth, and the temporal attention weights in Figure 10C are mainly concentrated in the shallow hidden layer states. The distribution of temporal attention weights indicates that the trajectory characteristics and joint velocities directly affect the temporal characteristics of manipulator dynamics.

**Figure 11:**
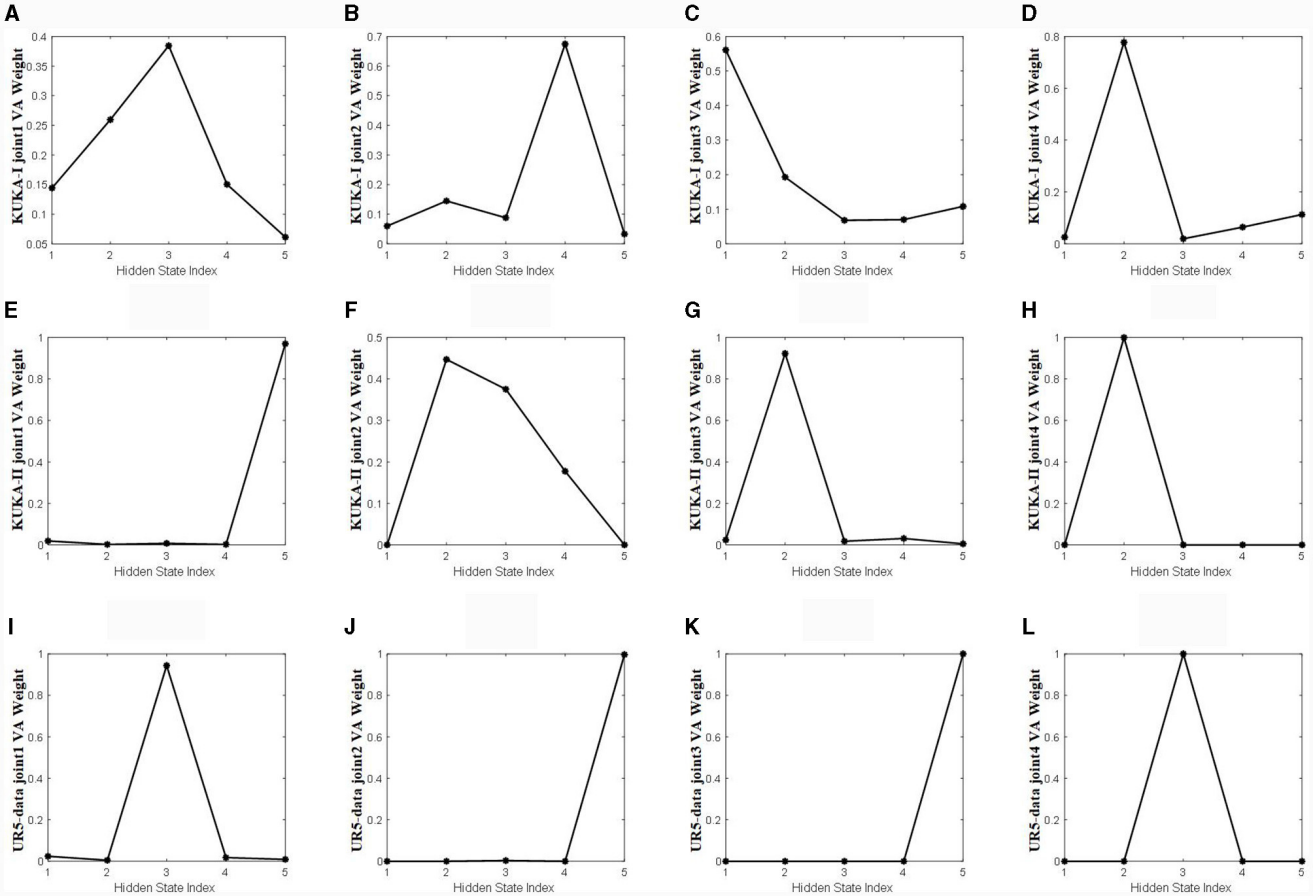
The temporal attention weights calculated by the velocity aware module. **(A–D)** Represents joints 1 to 4 in the KUKA-I dataset, **(E–H)** represents joints 1 to 4 in the KUKA-II dataset, and **(I–L)** represents joints 1 to 4 in the UR5-data dataset.

Figure 11 shows a visualization of the weights calculated using the velocity-aware temporal attention module proposed in this article. The weights are fused using the **TA-hn** and **TA-WA** described in Section 3.3. The weights are fused using the **TA-hn** and **TA-WA** described in Section 3.3. The vertical axis represents the weight value of **VA** calculated by the Velocity aware module, as shown in Figure 4C, and the horizontal axis represents the number of hidden layers. The VA weights in Figure 11 are attention weights calculated by VA-TA method. Combining Figures 10, 11 for observation, the more dispersed the weight of the time heatmap, the more dependent the weight calculated by the velocity-aware module on the modeling ability of **TA-hn**. The more concentrated the weight of the heatmap, the more dependent the weight calculated by the velocity-aware module on the modeling ability of **TA-WA**. This indicates that our velocity-aware method proposed in this article can better integrate the advantages of the two modes (**TA-hn** and **TA-WA**).

## 5 Conclusion

This article combines the spatiotemporal attention mechanism with the residual long short-term memory neural network to design an inverse dynamics model learning network for serial manipulators. A multi-layer perceptron spatiotemporal attention mechanism has been designed to reduce the impact of invalid input features on the model. We propose a velocity-aware LSTM hidden layer state fusion method, which weights and averages all hidden layer states of the LSTM, and then fuses them with the last hidden layer state. Two algorithms (**TA-hn** and **TA-WA**) are used complementary to concatenate the weighted average output and the final hidden layer output into a vector. The proposed fusion method fully utilizes the hidden layer states of LSTM networks, which is also a novel approach for time series modeling. The use of residual LSTM networks not only improves the convergence of the entire network but also solves problems such as vanishing gradients and exploding gradients. Finally, this article conducted ablation experiments with different configurations and obtained some patterns: Increasing the number of hidden layers of attention does not necessarily improve inverse dynamics model accuracy. Using the Sigmoid function for spatial attention and the SELU activation function for temporal attention can lead to better inverse dynamics models in different datasets. Residual connections help the network converge faster and improve the accuracy of the inverse dynamics model. Experimental comparisons are conducted on three datasets with other methods, and compared to the LSTM network, the proposed method improved model accuracy by at least 40% under the condition of no acceleration. Compared to other methods of state of art, the proposed method has the best modeling accuracy.

Through visualizing attention weights, it has revealed some patterns: when using spatial attention mechanisms to preprocess the input of the LSTM network, the positional information of the manipulator's joints is crucial to training a more accurate inverse dynamics model. Additionally, incorporating joint velocity information at the output of the LSTM network can effectively extract temporal features learned by the LSTM, preventing feature information loss and significantly improving model accuracy. These findings can predictably have a positive impact on future research of the manipulator inverse dynamics model.

## Data availability statement

The original contributions presented in the study are included in the article/supplementary material, further inquiries can be directed to the corresponding author.

## Author contributions

WH: Investigation, Methodology, Software, Writing – original draft. YL: Formal analysis, Supervision, Writing – review & editing. ML: Data curation, Software, Validation, Writing – review & editing. HM: Conceptualization, Funding acquisition, Supervision, Writing – review & editing.

## References

[B1] Baressi Šegota S. Andelić N. Lorencin I. Saga M. Car Z. (2020). Path planning optimization of six-degree-of-freedom robotic manipulators using evolutionary algorithms. International J. Adv. Robot. Syst. 17, 1729881420908076. 10.1177/1729881420908076

[B2] Çallar T. C. Böttger S. (2022). Hybrid learning of time-series inverse dynamics models for locally isotropic robot motion. IEEE Robot. Autom. Lett. 8, 1061–1068. 10.1109/LRA.2022.3222951

[B3] Cho K. Van Merriënboer B. Bahdanau D. Bengio Y. (2014). On the properties of neural machine translation: Encoder-decoder approaches. arXiv preprint arXiv:1409.1259. 10.3115/v1/W14-4012

[B4] Ding Y. Zhu Y. Feng J. Zhang P. Cheng Z. (2020). Interpretable spatio-temporal attention LSTM model for flood forecasting. Neurocomputing 403, 348–359. 10.1016/j.neucom.2020.04.110

[B5] Du Y. Yuan C. Li B. Zhao L. Li Y. Hu W. (2018). “Interaction-aware spatio-temporal pyramid attention networks for action classification,” in Proceedings of the European Conference on Computer Vision (ECCV) 373–389. 10.1007/978-3-030-01270-0_2334314355

[B6] Gautier M. Venture G. (2013). “Identification of standard dynamic parameters of robots with positive definite inertia matrix,” in 2013 IEEE/RSJ International Conference on Intelligent Robots and Systems (IEEE) 5815–5820. 10.1109/IROS.2013.6697198

[B7] Geist A. R. Trimpe S. (2021). Structured learning of rigid-body dynamics: a survey and unified view from a robotics perspective. GAMM-Mitteilungen 44, e202100009. 10.1002/gamm.202100009

[B8] Greff K. Srivastava R. K. Koutník J. Steunebrink B. R. Schmidhuber J. (2016). LSTM: a search space odyssey. IEEE Trans. Neur. Netw. Learn. Syst. 28, 2222–2232. 10.1109/TNNLS.2016.2582924 27411231

[B9] Karim M. M. Li Y. Qin R. Yin Z. (2022). A dynamic spatial-temporal attention network for early anticipation of traffic accidents. IEEE Trans. Intell. Transport. Syst. 23, 9590–9600. 10.1109/TITS.2022.3155613

[B10] Kong D. Wu F. (2018). “HST-LSTM: A hierarchical spatial-temporal long-short term memory network for location prediction,” in IJCAI, 2341–2347. 10.24963/ijcai.2018/324

[B11] Liu Y. Zhao C. Huang Y. (2022). A combined model for multivariate time series forecasting based on MLP-feedforward attention-LSTM. IEEE Access 10, 88644–88654. 10.1109/ACCESS.2022.3192430

[B12] Liu Z. Liu Q. Xu W. Wang L. Zhou Z. (2022). Robot learning towards smart robotic manufacturing: a review. Robot. Comput. Integr. Manufact. 77:102360. 10.1016/j.rcim.2022.102360

[B13] Liu Z. Peng K. Han L. Guan S. (2023). Modeling and control of robotic manipulators based on artificial neural networks: a review. Iranian J. Sci. Technol. Trans. Mechan. Eng. 47, 1307–1347. 10.1007/s40997-023-00596-3

[B14] Meier F. Hennig P. Schaal S. (2014). “Incremental local gaussian regression,” in Advances in Neural Information Processing Systems 27.

[B15] Mukhopadhyay R. Chaki R. Sutradhar A. Chattopadhyay P. (2019). “Model learning for robotic manipulators using recurrent neural networks,” in TENCON 2019–2019 IEEE Region 10 Conference (TENCON) (IEEE), 2251–2256. 10.1109/TENCON.2019.8929622

[B16] Nguyen-Tuong D. Seeger M. Peters J. (2009). Model learning with local gaussian process regression. Adv. Robot. 23, 2015–2034. 10.1163/016918609X12529286896877

[B17] Ostmeyer J. Cowell L. (2019). Machine learning on sequential data using a recurrent weighted average. Neurocomputing 331, 281–288. 10.1016/j.neucom.2018.11.066 30799908 PMC6380500

[B18] Polydoros A. S. Nalpantidis L. Krüger V. (2015). “Real-time deep learning of robotic manipulator inverse dynamics,” in 2015 IEEE/RSJ International Conference on Intelligent Robots and Systems (IROS) (IEEE), 3442–3448. 10.1109/IROS.2015.7353857

[B19] Reuss M. van Duijkeren N. Krug R. Becker P. Shaj V. Neumann G. (2022). End-to-end learning of hybrid inverse dynamics models for precise and compliant impedance control. arXiv preprint arXiv:2205.13804. 10.15607/RSS.2022.XVIII.066

[B20] Rueckert E. Nakatenus M. Tosatto S. Peters J. (2017). “Learning inverse dynamics models in o (n) time with lstm networks,” in 2017 IEEE-RAS 17th International Conference on Humanoid Robotics (Humanoids) (IEEE), 811–816.

[B21] Seeger M. (2004). Gaussian processes for machine learning. Int. J. Neural Syst. 14, 69–106. 10.1142/S0129065704001899 15112367

[B22] Sheng Z. An Z. Wang H. Chen G. Tian K. (2023). Residual LSTM based short-term load forecasting. Appl. Soft Comput. 144, 110461. 10.1016/j.asoc.2023.110461

[B23] Song S. Lan C. Xing J. Zeng W. Liu J. (2018). Spatio-temporal attention-based LSTM networks for 3D action recognition and detection. IEEE Trans. Image Proc. 27, 3459–3471. 10.1109/TIP.2018.2818328 29671746

[B24] Stathakis D. (2009). How many hidden layers and nodes?. Int. J. Remote Sens. 30, 2133–2147. 10.1080/01431160802549278

[B25] Tao F. Liu G. (2018). “Advanced LSTM: a study about better time dependency modeling in emotion recognition,” in 2018 IEEE International Conference on Acoustics, Speech and Signal Processing (ICASSP) (IEEE), 2906–2910. 10.1109/ICASSP.2018.8461750

[B26] Versaci M. Angiulli G. La Foresta F. Crucitti P. Laganá F. Pellicanó D.. (2022). “Innovative soft computing techniques for the evaluation of the mechanical stress state of steel plates,” in International Conference on Applied Intelligence and Informatics (Cham: Springer Nature Switzerland), 14–28. 10.1007/978-3-031-24801-6_2

[B27] Vijayakumar S. D'Souza A. Schaal S. (2005). LWPR: A Scalable Method for Incremental Online Learning in High Dimensions. Available online at: https://era.ed.ac.uk/handle/1842/3705 10.1162/089976605774320557 16212764

[B28] Williams C. Rasmussen C. (1995). “Gaussian processes for regression,” in Advances in Neural Information Processing Systems 8.

[B29] Xia L. Huang C. Xu Y. Dai P. Bo L. Zhang X.. (2022). Spatial-temporal sequential hypergraph network for crime prediction with dynamic multiplex relation learning. arXiv preprint arXiv:2201.02435. 10.24963/ijcai.2021/225

[B30] Xie Y. Liang R. Liang Z. Huang C. Zou C. Schuller B. (2019). Speech emotion classification using attention-based LSTM. IEEE/ACM Trans. Audio, Speech, Lang. Proc. 27, 1675–1685. 10.1109/TASLP.2019.292593437491423

[B31] Yilmaz N. Wu J. Y. Kazanzides P. Tumerdem U. (2020). “Neural network based inverse dynamics identification and external force estimation on the da Vinci Research Kit,” in 2020 IEEE International Conference on Robotics and Automation (ICRA) (IEEE), 1387–1393. 10.1109/ICRA40945.2020.9197445

[B32] Zhang S. Loweimi E. Bell P. Renals S. erqie (2020). “When can self-attention be replaced by feed forward layers?” arXiv preprint arXiv:2005.13895.

